# Nanoparticles and Controlled Delivery for Bioactive Compounds: Outlining Challenges for New “Smart-Foods” for Health

**DOI:** 10.3390/foods7050072

**Published:** 2018-05-07

**Authors:** MCarment Martínez-Ballesta, Ángel Gil-Izquierdo, Cristina García-Viguera, Raúl Domínguez-Perles

**Affiliations:** 1 Department of Plant Nutrition, Centro de Edafología y Biología Aplicada del Segura-Spanish Council for Scientific Research (CEBAS-CSIC), Campus de Espinardo 25, 30100 Espinardo, Murcia, Spain; mballesta@cebas.csic.es; 2 Research Group on Quality, Safety and Bioactivity of Plant Foods, Department of Food Science and Technology, Centro de Edafología y Biología Aplicada del Segura-Spanish Council for Scientific Research (CEBAS-CSIC), Campus de Espinardo 25, 30100 Espinardo, Murcia, Spain; angelgil@cebas.csic.es (A.G.-I.); cgviguera@cebas.csic.es (C.G.-V.)

**Keywords:** phytochemicals, nanoparticles, controlled release, improved functionality, health, smart-formulations

## Abstract

Nanotechnology is a field of research that has been stressed as a very valuable approach for the prevention and treatment of different human health disorders. This has been stressed as a delivery system for the therapeutic fight against an array of pathophysiological situations. Actually, industry has applied this technology in the search for new oral delivery alternatives obtained upon the modification of the solubility properties of bioactive compounds. Significant works have been made in the last years for testing the input that nanomaterials and nanoparticles provide for an array of pathophysiological situations. In this frame, this review addresses general questions concerning the extent to which nanoparticles offer alternatives that improve therapeutic value, while avoid toxicity, by releasing bioactive compounds specifically to target tissues affected by specific chemical and pathophysiological settings. In this regard, to date, the contribution of nanoparticles to protect encapsulated bioactive compounds from degradation as a result of gastrointestinal digestion and cellular metabolism, to enable their release in a controlled manner, enhancing biodistribution of bioactive compounds, and to allow them to target those tissues affected by biological disturbances has been demonstrated.

## 1. Introduction

In the late 1980s, Dr. Stephen De Felice first coined the term “nutraceuticals” that was defined as “as foods, food ingredients, or dietary supplements with demonstrated specific health or medical benefits, including the prevention and treatment of disease beyond basic nutritional functions” [1]. Nutraceuticals, in general, have been strongly suggested as candidates for the development of chemo-preventive agents, regarding several pathophysiological situations based in the experimental results retrieved from a range of studies [2]. Hence, upon the extensive research developed so far, some of their mechanisms of action that involve competencies to modulate multiple molecular pathways, without eliciting toxic side effects, have been demonstrated [1].

In recent years, and in close connection with the application of the promising chemo-preventive agents, technological advancements have allowed describing nano-biotechnology as a powerful tool to create and enhance the utility of nano-size materials [3], especially concerning the administration of bioactive phytochemicals in the frame of medical treatments [4]. In this respect, diverse types of nanoparticles have been tested for various uses. For instance, nano-silver has been widely used aimed to take advantage of its toxicity against a broad spectrum of microbes responsible, for instance, for superficial skin and nail infections in humans [5], in addition to oral and vulvovaginal corruptions [6]. Based on the recently gathered knowledge, one of the most relevant advantages of implementing this technology is to control the delivery of bioactive compounds, providing new and valuable responses to the current challenges for new “smart-foods”, able to promote human health and wellbeing. In this regard, this work reviews critically the recent advances on nanotechnology-related administration of bioactive compounds and its socio-economic implications.

### Societal Impact of Nanobased Technological Innovations for Food and Health

In recent years, nanotechnology has been noticed as one of the most important fields with clear socio-economic impact in several fields of activity. In this aspect, it has been estimated that the private sector has invested around 150 billion US dollars in the development of nanotechnology and application models during 2015. Regarding this, the number of products that incorporate nanoparticles in their formulations has enhanced, accounting for more than 1800 products containing nanomaterials, produced by 622 companies [7]. This new application alternative has been possible due to the 3.7 billion US dollars investment by USA, upon the National Nanotechnology Initiative, followed by the European Union and Japan, which have invested up to 1.2 billion and 750 million dollars, respectively, per year. If this evolution continues, in 2020, the worldwide economy could be supported by nanotechnologies, with an input of 3 trillion US dollars [8].

Nowadays, and regarding the Science and Technology of Foods, the application by the industry of the knowledge generated regarding nanotechnology is mainly focused on agriculture, foodstuffs transformation, packaging, and storage, and the production of dietary supplements [9]. Indeed, during food processing, the advances achieved so far have focused on nutrient delivery, considering distinct issues that deserve to be depth studied, such as the preservation of the physico-chemical features of foods (taste and color), and their safety, stability, bioavailability, and bioactivity. Regarding this, the use of new nanosystems might result in great interest to achieve additional application for the currently most widely characterized bio-functional compounds. Indeed, nanoencapsulation is a very valuable approach that could allow controlling bioactives’ release and their pathophysiological relevance, as well as their preservation after oral ingestion, with relevant applications for human health. As an example of such applications, structural lipids have been explored as carriers of healthy component applied for the inhibition of cholesterol transport from the digestive system to the bloodstream [10]. Thus, resorting to the research results, colloids, emulsions, biopolymers, liposomes, solid–lipid nanoparticles, and nanofibers have demonstrated an indubitable utility as vehicles of functional compounds [11].

Nonetheless, despite the increasing use of nanomaterials and the high number of derived commercial applications, nowadays, there are some concerns regarding the potential risk for human health derived from their use, as well as on the extent in which the inclusion of nanomaterials in foods formulation could compromise foods safety. Indeed, in this concern, one of the main matters of debate nowadays is focused on unravelling the extent to which nanoparticles and the bioactive compounds carried by them are competent to access to tissues naturally protected by biological barriers such as the blood–brain barrier [12,13], as well as the toxicological effects that this transportation could cause that have not been properly addressed so far.

Depending on the nature of nanoparticles, as well as the surrounding environment, the materials currently used can be aggregated, modifying their chemical properties, size, and shape [14]. These changes could result in an altered nanomaterial form and, in this sense, some reports have been focused on the evaluation of nanomaterials toxicity when they form part of a particular matrix [15]. To date, the gap in information existing in these concerns entails a lack of consensus about toxicity and direct/indirect consequences on health. As a result, the legislation governing the use of nanoparticles does not elucidate clearly this situation, being almost limited to the requirement of labeling food products containing nanomaterials. In 2009, the European Food Safety Authority published “The Potential Risks Arising from Nanoscience and Nanotechnologies on Food and Feed Safety” as an official document, while the European Union legislation differs depending if intended use of a specific nanocarrier is as a primary ingredient or constitutes a food additive [16]. However, the soft demand of the industry for incorporating this technology to production chains has prompted to a lack of a regulatory frame for the use of nanotechnologies in foods. In any event, to extend the utilization of this promising technology with industrial purposes, the advance of the desirable features of nanoparticles in diverse food matrixes constitutes an important challenge that deserves to be explored towards an enhanced use of such materials, according to their specific properties, at the time that contribute to preserve safety and bioactivity.

## 2. Biocompatible and Biodegradable Nanoparticles: Looking for the Best Option

### 2.1. Solid Nanoparticles as Attractive Drug Vehicles: Composition and Properties

A variety of nutrients and non-nutrients has been identified, regarding their biological activity once ingested by diet, being responsible for a diversity of biological activities, closely related with the promotion of human health. However, bioactive compounds from nutrients such as lipid derivatives (plant and mammals oxylipins), bioactive peptides, minerals and vitamins, as well as non-nutrients (phytochemicals), can be degraded as a result of the gastrointestinal digestion. Moreover, diverse barriers must be crossed by these compounds, which route towards the bloodstream, and the final distribution to cells and tissues, including their absorption at gastric or intestinal levels. During last decades, the search for effective carriers for bioactive compounds and drugs has been based on the identification of new alternatives for oral administration with health-promoting purposes, avoiding their early inactivation [17,18], because these factors limit the bioavailability of actives or drugs [19]. According to the results retrieved in recent years, nanoencapsulation developed using particles with diameters ranging from 1 to 100 nm enables augmented concentrations of bioactive compounds in cells and tissues, enhancing also shelf-life (reducing the metabolization and excretion of bioactive compounds) through a slowdown delivery [20]. Given these advantages, and aimed to optimize the benefits for health derived from nutraceutical formulations, regarding antioxidant, antiradical, and antitumor abilities, among others, the industry has boosted the research on this technology.

Upon evaluating the profits of implementing nanotechnology in agro-food industries, it has been noticed that nanoparticles have some advantages that deserve to be further explored. For instance, specific high surface zone of nanomaterials has been related to an enhanced number of functional groups, available for chemical reactions, relative to free functional compounds [14]. In addition, the class of relationships between nanocarriers and bioactive nutrients and non-nutrients, and the high area/volume ratios, contribute in some cases to suitable formulations, in which the adhesive properties of the nanocapsules led to prolonged transits of functional compounds through the gastrointestinal tract. This situation lets them reach unaltered specific areas, where their liberation provides enhanced benefits. Consequently, an augmentation of its absorption and bioavailability is achieved [21,22,23]. Moreover, additional constraints associated to the inclusion of bioactive compounds in the food matrix, such as the effect of light, temperature or oxygen, which compromise their stability and, thus, the bioactivity of the compounds of interest, can be attenuated using nanoparticles [24,25].

Among the nanometric size delivery systems, there are different types of encapsulation, which are classified depending not only on their nature, but also on the production method, properties of the system, the system free energy, the interaction force in the system, etc. [14]. To date, the most suitable nanoparticles with marked potential, regarding distribution of bioactive compounds, are lipid-based delivery systems, which may comprise solid–lipid nanoparticles, liposomes, and micelles, as well as protein- and polysaccharides-based biopolymeric nanoparticles. Specific structural properties of these nanosystems, as well as their low cost and non-toxic nature, make them suitable carriers in food delivery compared to others, such as synthetic polymeric nanoparticles [26]. In addition to nanoparticles, another nanotechnology application for the administration of bioactive compounds are nanoemulsion droplets, formed by dispersion or high-energy and low-energy emulsification methods. Although the main application of nanoemulsions is the preparation where droplets work as nanoreactors, nowadays new specific applications are being envisaged concerning controlled drug delivery and targeting [27].

#### 2.1.1. Lipid Nanoparticles

In the frame of nanotechnology, lipid-based systems are mainly represented by solid–lipid nanoparticles and nanostructured lipid carriers that have been commercially introduced as nanocarriers of functional compounds, in the last years, mainly due to their natural composition and biocompatibility. They can be made of different lipids, including fatty acids, steroids, and waxes to monoglycerides, diglycerides, and triglycerides [28]. A typical percentage ratio fat/aqueous medium of 0.1:30.0 (*w*/*w*) has been considered for developing solid–lipid nanoparticles [29] that feature spherical morphology and can be visualized, for instance, by transmission electron microscopy [30]. Particle size and stability can be affected by their lipid composition and the surfactant properties.

Within lipid nanoparticles, there are advantages associated to the use of liposomes that constitute nanosize artificial vesicles suitable to be obtained from phospholipids and cholesterol. In this concern, recently, the role of these vesicles as immunological adjuvants and drug carriers has been revealed [30,31]. Thus, it seems evident that several advantages can be obtained from using liposomes as carriers of bioactive compounds, including their capacity to encapsulate diverse bioactive compounds featured by a range of polarities, which are included into the aqueous core of the phospholipid vesicle or at the bilayer interface, and their structural versatility [32]. Besides, an interesting characteristic of liposomes is that they are obtained from natural lipids, being thus biodegradable, biologically inactive, and without antigenic, pyrogenic, or intrinsic toxicity [33,34]. The compounds within these nanoparticles are preserved from the deleterious activity of external factors, especially concerning enzymes and inhibitors present in the gastrointestinal tract [35]. Based on these benefits, liposomes are increasingly used by the pharmaceutical industry to control the release of several compounds of interest, because of their biological activity (drugs, vaccines, and enzymes) in the prevention of a range of pathological situations [32,36,37,38]. Nonetheless, liposomes are not absolutely free of drawbacks, as nanocarriers, due to their instability in plasma [39]. In this regard, nowadays, it is required to develop an evasion system to enhance the circulation time of these nanoparticles that would results in an augmented concentration in vascularized tissues [40], especially in cases involving active neoangiogenesis.

Apart from liposomes, micelles are very slim spherical additional lipid molecules of 10 to 100 nm formed in aqueous solutions [41]. These nanoparticles have been revealed as a valuable alternative to improve bioavailability and retention of bioactive phytochemicals, since they provide appropriate protection to these compounds against inactivation reactions by surrounding factors, and constitute an alternative featured by even higher loading capacity and improved stability [41,42]. The release of bioactive compounds from micelles is conditioned by an array of factors, including micelle stability, rate of bioactive compounds diffusion, the partition coefficient, the rate of copolymer biodegradation, the drug concentration within the micelles, the molecular weight, and the physicochemical features of the bioactive phytochemical and its location within the micelles [43,44]. Interestingly, release from micelles can also be enhanced in the targeted area by certain stimuli, such as pH, temperature, ultrasound, and light [45]. Thus, besides providing innovative solution to the solubility and long circulation of bioactive nutrients and non-nutrients, micelles contribute to a more efficient internalization and the proper localization within the separate cell compartments, where the biological activity of the compounds carried is required. This is extremely important because it helps take the highest advantage of the specific mechanism of action from the entrapped bioactive compounds [46], also contributing to higher concentrations at the organic site affected by pathological situations, thus decreasing side effects relatively to systemic administration [47].

Additionally, niosomes are microscopic lamellar structures formed upon non-ionic surfactant of alkyl or dialkyl polyglycerol ether class and cholesterol [48]. These lipid-based nanoparticles are structurally similar to liposomes, constituting an effective alternative to these carriers. Indeed, given their features as non-ionic particles, they seem to be less toxic, while providing an improved therapeutic index for bioactive compounds [49]. Moreover, the niosome vesicles are osmotically active and stable, and, thus, may act as a storage system, providing the controlled delivery of bioactive compounds. Thus, they augment oral bioavailability of compounds with limited absorption, allowing to envisage different therapeutic applications [50,51,52,53] and use as diagnostic imaging agents [49].

Solid–lipid nanoparticles share some features with nanoemulsions, such as low cost, good release profile, stability, scalable for industrial production, and food safety [54]. As described for nanoemulsions, when considering solid–lipid nanoparticles, the bioactive compounds are situated in the core of particle; however, higher stability and ability to withhold phytochemicals in the nanosystem complex has been described in relation to emulsions or liposomes [55].

Despite the promising features of solid–lipid nanoparticles, some potential disadvantages have been associated to these nanocarriers [55,56]. Regarding this, the loading capacity is one of the major constraints described so far. This seems to be a consequence of the crystallization or transformation of lipids. Hence, the use of nanostructured lipid carriers could be an alternative to overcome this constraint. Indeed, in nanostructured lipid carriers, although the lipid particles are also solid, the use of specific lipids, such as isopropyl myristate or hydroxyl octacosanyl, avoids crystallization at low temperatures [54].

#### 2.1.2. Polysaccharide Nanoparticles

In respect to biocompound delivery systems based on biocompatible materials, it is remarkable that those developed resorting to polysaccharides have been stressed as a valuable alternative to achieve controlled and prolonged release of bioactive phytochemicals [57]. In this aspect, the application of this type of compounds is reinforced by their amphiphilic nature that allows self-assembly in aqueous environments and helps to form specific structures [58,59]. Besides, polysaccharides feature a high affinity to mucosal cell layers in the respiratory and gastrointestinal tracts [60] that turns nanoparticles developed using these compounds into very valuable alternatives to enhance the bioavailability of the bioactive compounds of interest.

Based on the intrinsic charge of polysaccharide materials, they are classified into polyelectrolytes that include cationic, anionic and neutral subtypes, and non-polyelectrolytes [61]. Besides the intrinsic advantages noticed for this type of biocompatible material, it has recently been demonstrated that coat materials, applied on nanoparticles developed using polysaccharides, can interact with specific receptors in cells and tissues. The description of this interaction has allowed envisaging active and site-specific controlled targeting for compounds carried in polysaccharide nanoparticles [60].

Concerning cationic polyelectrolytes, chitosan, which is composed of repeated units of d-glucosamine, has been described as a non-toxic, biodegradable, and bioadhesive material that provides the advantages of control of release of encapsulated agents, complexation with negative charged macromolecules, avoidance of toxic solvents during preparation, and prolonged residence time at the site of absorption due to its mucoadhesive nature [62]. Indeed, the latter is one of the major advantages for the administration of compounds of interests against nasal, oral, ocular, and dermal disorders [63]. Several factors condition the preparation, conformation, and loading of chitosan nanoparticles, affecting their ability to delivery compounds [62]. In addition, chitosan nanoparticles are suitable to be modified by adding specific ligands that contribute to a more rapid and efficient interaction with cells membranes, and thus, to an enhanced delivery of bioactive compounds [64].

In respect to anionic polysaccharide nanoparticles alginate, heparin, pectin, and hyaluronic acid are important to mention [62]. An overview of the interest of these nanoparticles indicates that alginate features biocompatibility, biodegradability, non-antigenicity, and mucoadhesive features [65,66,67], which make it an interesting candidate for controlled delivery of bioactive compounds [62]. Regarding the mechanism of release, the electrostatic interaction between carboxyl groups and divalent ions mediates the formation of cross-linked gels, with a close relationship with the release of bioactive compounds [62], thus the data reported in the literature indicate these nanoparticles are a valuable alternative delivery system suitable for incorporating positively charged compounds. Interestingly, a combination of alginate and chitosan has been pointed out as an alternative system for the controlled delivery of bioactive compounds that could contribute to extend the circulation time, relatively to alginate and/or chitosan alone [62].

Apart from alginate, hyaluronic acid nanoparticles feature high aqueous solubility and stability, and are non-toxic and non-immunogenic, which have given rise to a reduced number of systems with potential on passive tumor targeting [68], since they exhibit affinity to hyaluronan receptors with a high expression pattern in tumor cells and, therefore, with high potential to carry bioactive compounds, specifically identified concerning anti-tumor activity, that could release in the precise site, contributing to increase their bioactivity.

Finally, in respect to neutral compounds, represented by dextran, pullulan, and pectin [62], the presence of hydroxyl groups modulates the incorporation of bioactive molecules in the base skeleton. Within this class of nanoparticles, dextran, for instance, has been used to design delivery systems able to escape the reticuloendothelial system, thus enhancing the circulation time of the bioactive compound of interest [62].

#### 2.1.3. Protein Nanoparticles

To date, diverse arguments have been proposed supporting the use of protein nanoparticles: (1) simple manufacturing by just heating protein solutions; (2) absence of requirement of emulsification performance; and (3) compatibility with the high pressure emulsification process. Moreover, protein nanoparticles feature high freeze–thaw stability, since the particles at the interface provide good stearic stabilization [69]. To date, different types of nanoparticles have been described, which are synthesized resorting to diverse classes of molecules. Actually, protein nanoparticles can be obtained with a plethora of lab approaches, each of them with its advantages and constraints. However, when compared with additional compounds suggested as nanoparticles for carrying bioactive compounds, protein-based nanoparticles have various advantages, namely the abundance of proteins in nature, their suitability to be transformed, and the absence of deleterious effects concerning the biological systems in which they are applied.

The first classification of protein-based nanoparticles refers to their origin (animal or plant proteins). The diverse types are associated to benefits and constraints, mainly regarding toxicity and/or infections associated to their application (animal proteins), while plant proteins provide the advantage of their hydrophobic characteristics because, which is associated with a lack of toxicity and a lower economic cost [70]. Besides, the surface features of protein-based nanoparticles are susceptible to be modified due to the occurrence of functional groups, which is of crucial relevance for obtained desired biodistribution, biocompatibility, molecules carrying capacity, and stability [70]. In addition, the surface amino groups, characteristics of proteins, allow the addition of hydrophilic polymers, such as polyethylene glycol that augment the circulation time [71]. The loading efficiency of protein-based nanoparticles is closely related to the isoelectric point. When assessing the compounds loading to the protein nanoparticles, it is noticed that the total amount of bioactive compounds is determined by monitoring (ultraviolet (UV)-spectrophotometry, fluorescence spectrophotometry or high performance liquid chromatography (HPLC)) the non-entrapped compounds present in the supernatants. This constitutes critical information for setting up the correct administration of the bioactive compounds. Additional features that should be considered regarding protein-based nanoparticles are the delivery of the compounds carried and the biodegradation of the nanoparticles, being both interdependent reactions [69].

#### 2.1.4. Nanoemulsions

As mentioned before, the application of nanoemulsion droplets, to the controlled delivery of bioactive compounds, is a recent approach in the field of Food Science and Technology, thus still has much further to go [27]. To date, nanoemulsions have been mainly applied to the design of new functional foods, since this technology allows regulating the release of bioactive nutrients and non-nutrients featured by poor water solubility [72,73]. For the preparation of nanoemulsions, aqueous solutions of lipid droplets (size approximately 100 nm) are prepared using different methodologies. The most common technology applied is high pressure homogenization [74,75] that avoids particle aggregation and compounds separation by gravitation in comparison with normal emulsions [76]. In fact, in nanoemulsions, the functional compounds are protected from reacting with the food matrix, which helps to preserve organoleptic properties, while enhancing the bioavailability of the bioactive compounds of interest, because of favoring passive transport through biological membranes [77,78]. Afterwards, during digestion, it has been reported that nanoencapsulation increases solubility of bioactive nutrients and non-nutrients, as well as their presence suiting aborbtion at the gastric and intestinal levels. Moreover, the application of these compounds using nanoemulsions provides enhanced protection, reducing their metabolism and lowering the activity of efflux transporters [79,80].

Currently, there is an extensive literature on the encapsulation of functional compounds, such as curcumin and resveratrol [73,81,82,83], using nanoemulsions to protect these compounds from digestion. Indeed, despite the evidence retrieved through the last decades from the array of in vivo and in vitro characterizations, developed on the benefits for human health of bioactive phytochemicals, their current use is still limited due to poor bioavailability [84]. Regarding this constraint, an adequate formulation of nanoemulsifier has been demonstrated as a valuable alternative to improve the protection of these compounds from chemical and enzymatic aggressions in the intestinal lumen, and thus provide an enhanced absorption and bioavailability. In this sense, combinations of lipophilic and hydrophilic emulsifiers, specifically nanoemulsions composed by lecithin, are competent to entrap resveratrol in the nanoparticle core by hydrophobic forces, enhancing not only the shelf-life of the bioactive molecules, but also their bioavailability by increasing the intestinal absorption and cellular uptake [85].

In addition to the specificity of the nanoemulsions for the diverse bioactive compounds, their stability is also a critical issue that has been deeply evaluated in recent years. Hence, the stability during storage is conditioned by an array of factors, namely pH, temperature, and ion strength, but also on some intrinsic properties of the formulation as the emulsifier concentration or the use of additives that should be adjusted carefully to guarantee the appropriate functionality of nanoemulsions [75,86].

Another factor that has to be considered to achieve the best outcomes from nanoemulsions, as carriers of bioactive compounds, is the nanoparticles size. The right nanosize of emulsions could be reached using co-adjuvants, since droplets prepared from low viscosity oils have been shown to be able to reach the nanoscale. An example of this is the addition of butanol to sodium caseinate that presents a nanoscale after the addition of the alcohol [87]. Polyethylene glycol and ethylene glycol have also been used to achieve the desired nanomagnitude.

From the technological point of view, in the beverage industry, an additional quality of nanoemulsions, i.e. the high optical clarity, provides advantages during manufacturing that have advised on the challenge of prioritizing this technology in the design of new added-value products [88]. However, it is required to state that each protein has the capacity to encapsulate specific compounds according to their hydrophobicity or hydrophilic features.

### 2.2. Nanotechnology for Medical and Nutrition Research

As mentioned above, dietary supplements are useful for the prevention or reduction of different pathophysiological situations and diseases, supported by their antioxidant, anti-inflammatory, and antitumoral properties, among others [89]. Encouraged by the extensive bibliography on these biological activities, many supplements have included phytochemicals in their composition. Given diverse troubles reported regarding bioavailability (that is closely linked to their biological activity in vivo), to take advantage of their biological potential, research efforts have been addressed to explore the diverse encapsulation modes that would enhance bioavailability. Although this approach has allowed developing new nanoencapsulated products addressed to be administrated via ocular, transdermal, and intravenous [90,91], their application in the design of functional foods, because of the specific chemical and enzymatic conditions in the gastro-intestinal compartments, with a direct impact on their release from the food matrix after oral ingestion, integrity, and functionality, needs to be further explored [92].

As an example of these applications, nanoemulsions have been successfully used for curcumin (diferuloylmethane) and dibenzoylmethane (a structural analog of curcumin) encapsulation [93]. These compounds have demonstrated valuable therapeutic potential, while exerting reduced toxicity, in phase I human clinical trials [94]. Upon the studies developed to improve their bioavailability and bioactivity, it has been found that using a mix of triacylglycerol (as oil) and Tween-20 (as emulsifier) could be enhanced the cellular uptake of curcumin and, consequently, an amended anti-inflammatory activity [93,95]. Hence, although other encapsulation alternatives have been considered for curcumin, such as hydrophobic starch [96], albumin [97], β-lactoglobulin [98] or chitosan [99], the poor water-solubility of these compounds limited their application, remaining nanoemulsions as the most suitable and efficient encapsulation alternative.

Other polyphenols of interest have been nanoencapsulated in emulsions for experimentation, such as tannin, stilbenes, and flavonoids [100]. This reinforces the idea of nanoemulsions as a valuable choice for these types of bioactive compounds. However, the emulsion composition has been reported as a decisive factor for the final bioactivity. Thus, epigallocatechin gallate, a hydrophilic flavonol, featured by similar and low bioactivity as antioxidant, in mice tissues, when formulated in two esterified emulsions or in an aqueous phase, while the selection of a third emulsion composition results in an increased antioxidant activity in mice plasma [101].

Essential oils are other important plant derived bioactive compounds with interesting benefits, regarding the prevention of chronic pathologies or infectious diseases. Indeed, based on this evidence, these compounds have been applied as analgesic, sedative, anti-inflammatory, spasmolytic, and local anesthetic treatments [102]. Thus, although a great variability in micro encapsulation methods has been described for these compounds, such as polysaccharides, spray-dried powders or Ca-alginate [103,104], these encapsulation alternatives provide protection to essential oils but seem to be no competent to enhance their antimicrobial activity. However, the nanoscale promoted a slower delivery and higher cell permeability, especially in the skin layers, increasing the activity of essential oils [105].

According to the information referred above, lipid-based nanocarriers are noticed as valuable alternatives to entrap essential oils in the core of the nanostructure and thus, reach different type of cells. In this regard, these nanoparticles have been applied in microbial infections due to their higher cell penetrability [106]; however, in some cases, antimicrobial capacity was reduced when compares with microencapsulation forms [24].

Efficient nanoencapsulation of other compounds, such as ferulic acid and tocopherol in solid–lipid nanoparticles, has been also reported recently [107]. Thus, in nanoemulsions, bioactive lipids were protected against autoxidation [108,109] and carotenoids [110], increasing their bioaccesibility.

Another functional compounds used in the formulation of health foods are probiotics, microorganisms, that improve intestinal microflora. Regarding probiotics, targeting specific regions in gastrointestinal tract after nanoencapsulation has been achieved [111]. Nanoemulsions were used to protect lactic acid bacteria [112] and, thus, to fine-tune the microbiota in the diverse intestinal conditions.

The micelle delivery system, for instance, is widely accepted as valuable carriers of antitumor drugs, and has been evaluated in the frame of clinical trials [43]. In this regard, hydrophobic drugs, for instance, can only be administered intravenously after their conditioning with solubilizing adjuvants, which has been associated with the appearance of toxic symptoms [113]. However, the addition of these drugs into nanocarrier-like micelles allows the replacement of the toxic adjuvants and contributes to minimize toxic effects [114]. Furthermore, as an additional advantage, the unimolecular micelles exhibit a maintained distribution jointly with a controlled pH-sensitive drug release. Likewise, it has been demonstrated that rapamycin could be loaded efficiently in mixed micelles up to a concentration of 1.8 mg/mL by using a hot-shock protocol. Upon these characterization, the release kinetic of obtained for rapamycin indicates that this micellar system could be triggered by varied pH environments under physiological conditions [115]. Matsumura et al. demonstrated that a paclitaxel micellar formulation consisting of polyethylene glycol and modified polyaspartate as hydrophobic block features cytotoxicity in a range of human tumor cell lines compared with the administration of the bioactive compound alone [116].

## 3. Phytochemicals Loaded Nanoparticles: Diving into Nanosized Drug Delivery Systems

### 3.1. Bioavailability Advantages of Nanoencapsulated Phytochemicals

Phytochemicals feature a diversity of chemical structures, represented by phenolic acids, indoles, alkaloids, isothiocyanates, phytosterols, saponins, and phytoprostanes/furanes [117,118]. These compounds have received growing attention boosted by their benefits for human health [119,120], upon a plethora of mechanisms including free radicals scavenging, inhibition of the assembly of microtubule and microfilament, metal chelation, and/or protease inhibition, among others [121]. Hence, the assessment of these compounds, from their in vivo metabolization point of view, has revealed that, after oral intake, phytochemicals are recognized and processed as xenobiotics and degraded as a consequence of the different digestion chemical, enzymatic, and microbial phases in mouth, stomach, and small and large intestine; then absorbed, distributed, and metabolized in cells and tissues; and finally excreted via renal, biliary or pulmonary [122]. Meanwhile, the maximum bioaccessibility and bioavailability of phytochemicals give rise to their actual capacity to exert biological effects in vivo (bioefficacy). The bioactivity of the bioactive nutrients and non-nutrients is demonstrated by short-term changes in the expression of biomarkers of liver function, plasma lipid profiles, blood pressure, plasma glucose, and plasma antioxidant activity [123]. In this sense, bioefficacy is influenced by the chemical nature of these compounds and their metabolic derivatives.

The innumerous articles published to date on the bioavailability of phytochemicals thereafter their oral intake have demonstrated that these compounds or their metabolites may fall in the nano/picomolar range in cells and tissues, which may result in insufficient dose to achieve the biological efficacy, demonstrated upon in vitro characterizations [124].

The dispersion and absorption, of these compounds, is closely dependent on their polarity. Once absorbed, small intestinal enterocytes are responsible for the cellular uptake, efflux pumping, and phase I and II metabolism, while the results of these processes are responsible for the amount of bioaccessible phytochemicals [122]. Moreover, transformations occurring in the frame of phase I and II reactions in hepatocytes give rise to active formats from inactive precursors that are the actual responsible of their biological value [125]. In addition, unabsorbed phytochemicals reach the large intestine, where are further metabolized by the local microbiota. This additional modification also affects their bioefficacy, providing new bioactive compounds that contribute to the final biological interest of a given plant matrix [122].

To prevent the deleterious effect of metabolization on the occurrence of truly bioactive compounds in cells and tissues, at operative concentrations, it has been pointed out that edible nanoencapsulation vehicles could be a valuable alternative. Regarding this, nanoencapsulation technology has been implemented, based on the information retrieved from an array of in vitro and in vivo characterizations, set up aimed at assessing the capability of different tissues and cell types to uptake the bioactive compounds at operative concentrations, according to an array of pathophysiological situations.

Diverse safe ingredients including lipids, polysaccharides, proteins, and biodegradable polymers are currently applied towards the development of edible nanoparticles featured by well-characterized size, surface properties, matrix materials, and compartment structure. These nanoparticles are developed foreseen to obtain improved strategies to enhance the release of the compounds of interest in target cells and tissues, as well as an increased bioefficiency [126,127]. The diverse types of nanoparticles suitable to be used as carriers of bioactive phytochemicals, in the frame of oral administrations, display a variety of structural features, such as is exemplified by nanoliposome, micelle, nanoemulsion, solid–lipid nanoparticles, polymeric nanoparticles (nanospheres and nanocapsules), protein–polysaccharide complex coacervation, cyclodextrin inclusion, and polymeric nanogel. Indeed, these alternative nanoparticles provide a range of features, including the process of loading phytochemical compounds, encapsulation efficiency, stability during gastrointestinal digestion, the releasing mechanisms and, therefore, their capacity to enhance the bioefficacy of the carried phytochemicals [122].

This situation has been recently illustrated by the evaluation of neuroblastoma cells (SH-SY5Y cell line), on their ability to uptake nanoparticles that provide promising results. In fact, the results retrieved from this work revealed that nanoparticles contribute to reach efficient concentrations of the bioactive compounds in the nervous system, especially when applying anionic charged nanoparticles, that exert a higher capacity to successfully go-through the blood–brain barrier, in comparison with cationic nanoparticles [128]. In addition, when using this type of nanoparticles, given the repulsion among the high negatively charged nanoparticles, it is possible to achieve extra stability for the bioactive compounds in cells and tissues, in vivo [129].

A careful revision of the literature gives a close relationship between the capacity of nanoparticles to enhance phytochemicals’ bioefficacy with particle size (the most relevant feature), surface properties, matrix materials, and compartment structure [130]. Actually, these features affect bioefficacy, influencing dispersion and gastrointestinal stability, as well as the release rate and the delivery site, the efficacy of the transportation through the endothelial cell layer, the systemic spread, and their capacity to control the impact of the microbiota metabolism [122]. Besides, when targeting the improvement of cellular uptake, it is essential to notice that particle size, jointly with the use of polymeric nanoparticles, solid–lipid nanoparticles, or nanoemulsions, contribute to the stability by affecting to the repulsion facts and/or interfacial-tension-decreasing compounds [131,132], which contributes to augment cellular uptake.

Based on these advantages, loading phytochemicals within such edible nanoparticles enhances the dispersion of initially water-insoluble phytochemicals in aqueous media [133], while the physical barrier provided by nanoparticles protects bioactive compounds against oxidation under acidic and alkaline degradative conditions, in stomach and small intestine, respectively [134,135].

In the literature the bioactivity of phytochemicals in their native state has been frequently reported, while, in vivo, as mentioned before, the delivery of bioactive nutrients and non-nutrients are critically affected by their solubility, which affects stability and penetration under gastrointestinal conditions. To overwhelm this conflict is a basic objective of nanotechnology in the field of Science and Technology of Foods, specifically regarding the development of functional foods and nutraceuticals. Hence, encapsulation of phytochemicals constitutes valuable alternatives to release bioactive compounds in specific tissues, affected by pathophysiological situations. In this sense, carrying bioactive compounds on nanoparticle limits their degradation under the chemical and enzymatic conditions of the gastrointestinal tract, as well as their general metabolism. In this regard, the physico-chemical features of the materials used to develop nanoparticles, as well as the digestibility of nanoparticles in the gastric or intestinal sections, are key issues that should be addressed, firstly, when designing a nanoparticles-based strategy to the oral administration of phytochemicals [122].

Some examples illustrate this situation: while starch-based nanoparticles are digested at oral level by the activity of α-amylase, additional polysaccharide nanoparticles are degraded in the small intestine and protein−polysaccharide nanoparticles can be due to variations of pH and salt concentrations, such as those occurring in the gastrointestinal lumen during the separate stages of digestion [135]. Lipid nanovesicles release phytochemicals in the small intestine simultaneously to the digestion of triglycerides [122].

In addition to the nature of the nanoparticle, the loading method has been revealed to be critical for the final release of phytochemicals and, thus, for their bioavailability and bioactivity. Regarding this, the drug-loading method strongly influences the loading efficiency, and it has been described that nanoencapsulated compounds release to a lower extent relative to nanosphere-based delivery, even if both formulations have similar efficiency [90].

Recently, a new type of compounds has emerged, plant oxylipins, such as phytoprostanes and phytofurans, which are formed by non-enzymatic oxidation of α-linolenic acid. Bioavailability and biological activity of such compounds have been suggested based on their structural analogy with human eicosanoids [118,136]. These lipid phytochemicals are compounds of special features as they migrate into fatty acids-based micelles, which contribute to their solubility and subsequent cellular transportation [122].

Apart from the control of phytochemicals release, nanoparticles also influence the transport of bioactive compounds through enterocytes (by transcellular endocytosis for particles between 20 and 1000 nm), by modulating residence time, transportation efficiency, and pathways, as well as the metabolic reactions undergone by such compounds within endothelial cells. Hence, for instance, chitosan derivatives have been related to mucoadhesive features and their capacity to open tight junctions, improving paracellular transport [137,138,139]. Moreover, the nanoparticle’s charge also influences the formation of hydrogen bonds with the mucosal surface, contributing to a momentary retention [140]. Another feature of nanoparticles that influences their role as carriers of bioactive compounds is the incorporation of cell-penetrating ligands that could contribute to enhance transmembrane transport efficiency [122].

Once absorbed from the intestinal lumen, nanoparticles loaded with bioactive phytochemicals move through endothelial layer to migrate to tissues into bloodstream. At this stage, nanoparticles have already been included in endosomes and undergone oxidation, reduction, and hydrolysis reactions, among others, as well as conjugations by diverse metabolization routes upon phase II metabolism. However, unlike metabolism of free phytochemicals, when carried on nanoparticles, the bioactive compounds are protected from such reactions, by modulating the exposition to metabolizing enzymes in enterocytes [141].

Highly lipophilic phytochemicals featured by specific features, regarding the logarithm of the oil−water partition coefficient and a long-chain triglyceride solubility (e.g., phytoprostanes and phytofurans), may go through enterocytes and form chylomicrons with enterocyte lipoproteins [142]. These reach the bloodstream via mesenteric lymph and thoracic ducts, avoiding hepatic first-pass metabolism. This route of absorption has been imitated using nanoparticles incorporating a lipid phase (for instance, emulsions, liposomes, and solid–lipid nanoparticles), which enhance the participation of the lymphatic absorption at the time that augment the rate of transcellular absorption [143]. Given the interest of integrating lipid phase in nanoparticles as modulators of the absorption rate, the extent to which the phospholipid composition and concentration of emulsifiers are competent to fine-tune biodistribution by modifying the lipophylicity and plasma-binding properties of phytochemicals has been investigated [144].

Additional approaches that have been explored, as valuable modes to control release and bioavailability of bioactive nutrients and non-nutrients administered, using nanoparticles as carriers are represented by the use of magnetic nanoparticles, the alteration of the surface chemical properties, and the inclusion of ligands facilitating the cellular uptake [122]. However, for some types of compounds, such as stilbenes, flavonols, and anthocyanins, which are metabolized by the intestinal microbiota towards even more powerful bioactive compounds, could be interesting for avoiding their digestion and absorption at gastric and small intestine levels. With this objective, the best option lies in the use of nanoparticles manufactured with materials or multilayer structures, resistant to gastric and small intestine conditions in terms of pH, osmotic conditions, and enzymatic activities. In addition, to achieve this objective, it is required to design release mechanisms that allow the delivery of bioactive phytochemicals under the osmotic and pH conditions of the large intestine, in which the microbiota responsible for its transformation is present [122]. On the other hand, once this objective is achieved, the challenge of reaching highly bioavailable compounds, derived from the microbiota metabolism, constitutes an additional constraint, due to the low colonic absorption at the conditions of luminal fluid volume, viscosity, and neutral pH of colon [145,146].

Once absorbed and circulating in blood stream, diverse strategies have been suggested and explored to control the length of circulation, such as using hydrophilic coating. Polyethylene glycol has been demonstrated as reliable to enhance circulation time, by acting as a steric barrier and thus protecting nanoparticles from opsonization [147].

One of the most widely used material for the development of nanoparticles is poly-(lactic-coglycolic acid) that represents a successfully used biodegradable polymer due to the production of the metabolite monomers lactic acid and glycolic acid during their physiological hydrolysis, that are easily processed via the Krebs cycle. Due to this rapid metabolization, low systemic toxicity has been described associated to the application of this biomaterial for the control of the bioactives compounds release [148]. However, it is crucial to develop surface modifications using nontoxic and blood compatible material, that allow their uptake by macrophages and the augment of the length of blood circulation, which contribute to the sustained delivery of the bioactive compounds carried in this nanosystem [149,150]. In this connection, poly(ethylene-glycol) is used as hydrophilic nontoxic segment in combination with hydrophobic biodegradable aliphatic polyesters, because this provides the capacity to resist against opsonizing and the ulterior phagocytosis, contributing to enlarged shelf life in the bloodstream and tissues [151,152,153].

### 3.2. Nanotechnology for Bioactives Delivery

In recent years, nanotechnology has emerged as a valuable alternative to control drugs delivery and tissue engineering [31], because of the chance provided by this to design and develop new strategies that could enhance the efficacy of natural bioactive molecules, currently used in a very limited extent, as therapeutic compounds for the treatment of different pathophysiological situations [32]. Indeed, nanotechnology is an alternative to traditional formulation, which could contribute to improve general bioavailability and site-specific release, simultaneously to toxicity reduction [33,34,35]. According to these aims, several epidemiological studies have been developed, during the last years, aiming to take higher advantages of the influence of dietary habits on the incidence of an array of pathophysiological situations, that have focused the assessment of several plant foods. From such work, multiple compounds that display potential health benefits in vitro have been identified [154,155]. However, when evaluating the activity of those compounds upon in vivo studies, many of them were not competent to translate the activities previously demonstrated in vitro, appearing as unstable in the intestinal environments and, thus, exhibiting poor bioavailability. Thus, to achieve the required bioavailability rates to take advantage of their biological activities, as a first approach, higher doses were tested, which showed efficacy but resulted in systemic toxicity [95].

The major outcomes from these studies have allowed to notice that several factors are involved in bioavailability, viz. chemical structure, solubility, stability against gastric and colonic pH, metabolism by gut microflora, absorption across the intestinal wall, active efflux mechanism, and first-pass metabolic effects [147]. However, when reviewing the data reported on the bioavailability of phytochemicals, it is revealed that many of them are only partially absorbed/metabolized by the intestinal epithelium, a cellular type responsible for systemic occurrence by their uptake using efflux transporters (mainly P-glycoprotein) [156]. The efflux transporter proteins mediate the active extrusion of compounds back into the intestinal lumen [157] that, in turn, limit the bioavailability of the bioactive phytochemicals of interest to nanomolar concentrations in blood, even if the ions have been detected the intestinal section, where they are absorbed, in high concentrations [158]. Indeed, this situation demonstrates that it is difficult to assess the bioavailability of chemical agents based solely on their physicochemical properties.

This is not just a general concept related to the biological activity of a given phytochemical compound, the bioavailability of the compound itself, at the target site, is a major issue that has not be appropriately addressed in the last years [159]. In this regard, understanding the concerns enclosed to the bioavailability of an individual compound is essential to draw strategies that might help to overwhelm these limitations. In this sense, the emergence of new technologies that allow the advance towards efficient and safe administration of newly identified bioactive compounds, has increased the interest in new “smart” delivery systems that contributes to improve the pharmacological properties of the administrated compounds [147]. Hence, a promising approach to avoid low bioavailability and systemic toxicity, associated in some extent to the use of xenoparticles as carriers of the bioactive compounds of interest, is the application of nanoparticles manufactured using materials of demonstrated safety, such as polymeric nanoparticles, liposomes, dendrimers, and micelles [160,161]. Actually, the use of these types of carriers is associated to innumerous advantages relatively to the traditional systemic administration, since this approach constitutes a powerful tool to modulate the pharmacokinetics and to improve delivery of bioactive agents to target sites [147].

Indeed, nanoparticles may enhance the oral bioavailability of poorly soluble compounds, as well as the tissue uptake after parenteral administration, supplying enhanced adherence capacity to the capillary wall during the initial diffusion phases (tethering, rolling, adhesion, and transmigration). Moreover, the selection of the most appropriate options between the currently developed could also allow augmenting the delivery across membranes and biological barriers [162]. In this regard, the size limitation to go through diverse biological barriers is closely related to target location and the chemical features of the tissue [163].

In recent years, stimuli-responsive polymer-based nanocarriers have focused the attention of researchers, working on the evaluation of bioactive phytochemicals in vivo. Regarding this, such nanoparticles are exposed to modification of their physical and chemical properties when exposed to the specific conditions (pH, temperature, light, magnetic field or glucose levels) in the intestinal tract or the diverse tissues after distribution [163,164]. This dependency could be of significant interest, as they provide the chance to set up valuable relationships between the release of bioactive compounds and specific niches or pathological state [132]. This situation is exemplified by paclitaxel delivery once loaded on pH-responsive nanoparticles, which has evidenced its utility upon in vitro and in vivo antitumoral determinations [165,166].

Anthocyanins have been highlighted, for several years, as model phytochemical to assess the potential of competence of polymeric nanoparticles to encapsulate hydrophilic compounds. The final objective of these studies is to establish the contribution of these approaches to the bioavailability and controlled release and release kinetics in vitro. In the case of anthocyanin, these features have been evaluated on polyethylene glycol moieties. Hence, in these works, anthocyanins were encapsulated with 60% efficiency in biodegradable nanoparticle formulation based on polylactide-*co*-glycolide, which were stabilizer resorting to the use of polyethylene glycol. These nanoparticles evidenced a biphasic release profile, in vitro. In addition, all the polyethileneglicosylated nanoparticles present a similar delivery pattern, characterized by an initial abrupt release followed by a continued supply [167]. Hence, this work described that bioactive compounds may be adsorbed on the surface of nanoparticles as a burst release that is not maintained in time, while the sustained release is due to the liberation of compounds encapsulated in the core domains of the nanoparticle [168].

### 3.3. Nanoparticles towards Targeted Bioactivity

A further step of what has been discussed above is the exploration of the advantages provided by nanotechnology concerning the release bioactive compounds carried in nanoparticles in specific tissues or cell types affected by pathophysiological events that needs to be treated. In the last decades, it has been boosted the application of bioactive phytochemicals as promising compounds capable to prevent health disturbance by modulating molecular pathways in cells. These compounds feature strong radical scavenging, anti-inflammatory, and neuroprotective activities, among others. Nonetheless, most bioactive nutrients and non-nutrients are unstable and rapidly transformed into other compounds with different biological attributions, which jointly with their fast degradation and excretion, limits the actual benefits that could be retrieved from such healthy compounds [169]. For instance, during digestion, the hydroxyl radicals of phenolics (for instance, anthocyanins) are oxidized into quinones, reducing the biological power of these molecules [170]. Traditionally, these constraints have been overwhelmed by therapeutic alternatives outlined for several diseases, which include invasive in situ administration of bioactive drugs, as a way to guarantee high tissue concentrations of the functional molecules of interest. Nevertheless, these administration alternatives are characterized by reduced patient compliance, because of the distress associated with frequent administrations, generally related to an array of side effects [171,172]. Alternatively, substitutive application forms of bioactive phytochemicals have been associated to augmented costs, particularly when repeated administrations are required to complete the treatment for a specific pathophysiological situation [173].

Apart from the side-impacts of the traditional administration of bioactive compounds, a loss of bioactivity during storage, due to the xenobiotics metabolism in mammals, has recently been reported, which could prevent them from reaching the target tissues and cells in which their activity is desired [174,175,176].

To date, diverse works have been developed aimed to identify alternatives that allow to overcome these problems; for instance, by using high dose or multiple treatments of bioactive compounds. However, this has been associated with dangerous side effects related to overdoses due to nonspecific toxicity of such compounds [174]. As a result, it has been identified an urgency to develop biocompatible and biodegradable systems for packaging phytochemicals, based on carrier systems constituted, for example, by oil-in-water emulsions and liposomes [177]. These alternatives have been demonstrated useful to provide sustained release of therapeutics in target tissues and, in addition, to reduce biological disturbances in cells and tissues, no compromising the stability and functionality of macromolecules [178]. Hence, this provides stability that in turn is responsible for a higher bioavailability and long term circulation [178], due to the protection supplied against outer stresses in vivo [179]. The research on controlled delivery options in respect to tissue, concentration, and pathophysiological modifications, has given rise to the description of biodegradable polymers and formulations specific for diverse types of bioactive compounds, with interesting biological activities, which have focused the preparation of phytochemicals-encapsulated nanoparticles [180]. Based on these applications, this technology has been noticed as highly useful for controlled delivery of bioactive compounds in target sites.

Moreover, the structural features of the nanoencapsulations pointed out so far, have a direct impact on the release profile [90,181]. This is specifically referred to as core−shell nanoparticles, double-emulsions, gelled networks, multiple coating systems, and “prodrug delivery systems”. Actually, when these systems are conjugated with azo- or glucuronic acid-including polymers, it is possible to control the bioactive compounds carried by enzymatic hydrolysis, which can contribute to retard the speed of phytochemicals release, as well as a noticeable change of their physical dispersion state [123].

This is of special relevance regarding compounds that develop their biological functions in tissues with high protection against xenobiotics by biological barriers, such as the blood–brain barrier. In this frame, anthocyanins contribute to improve brain functionality and to reduce oxidative stress associated to normal cells metabolism, resorting to their radical scavenging, anti-inflammatory, and anti-neurodegenerative capacity [168,170], thus preventing memory losses in estrogen-deficient rats [182]. The polymer-based nanoparticles used for characterization in this model were featured by a biphasic release profile, providing enhanced neuroprotective power of anthocyanins against Alzheimer’s dementia. Besides, the efficiency of the anthocyanins administered associated to nanoparticles has been further demonstrated by monitoring the capacity to attenuate the expression of clinical (amyloid precursor protein and beta-site amyloid precursor protein cleaving enzyme-1), inflammatory (p-nuclear factor kappa-B, Tumor Necrosis Factor-alpha, and nitric oxide synthase), and apoptotic (B-cell lymphoma-2 (BCL2), BCL2 associated X (Bax), and caspase-3 protein) markers [170]. Using this model, it was evidenced that anthocyanins loaded nanoparticles reduced significantly the level of protein markers and were also more efficient in modulating the P38/JNK pathway, according to reduced expression of various inflammatory markers, cytotoxic compounds, and proinflammatory cytokines [168]. In respect to oxidative stress, non-conjugated anthocyanins or molecules associated to nanoparticles significantly upregulate endogenous antioxidant genes, such as nuclear respiratory factor-2 and HO-1 proteins, which has been related with the prevention of oxidative stress and consequently, with an attenuation of the clinical symptoms of the Alzheimer’s dementia [183].

An additional functionality demonstrated for anthocyanins loaded nanoparticles is related with their capacity to revert the augmented and decreased expression of pro-apoptotic (caspase-3 and Bax) and anti-apoptotic proteins (BCL2), respectively, thus reducing DNA damage in a higher extent than native non-conjugated anthocyanin. All these findings together prompted Amin et al. [168] to suggest a neuroprotection activity of anthocyanins loaded nanoparticles that surpassed significantly the biological potential, enclosed to free anthocyanin. This enhanced activity, when combined with polysaccharides, seems to be related to an increased stability and prolonged degradation time [170].

## 4. In Situ Bioactive Compounds Delivery Control: Drawbacks and Breakthrough Advantages

According to the constraints outlined before, in the last decades, growing attention and resources have been devoted to the development of carrier systems that allow local delivery of therapeutic agents, for instance, by using organic materials. Regarding this, control delivery has been identified as a key stage of bioactive compounds administration, foreseen as a way to set up the timing, tissue/cell, and pathophysiological conditions, under which therapeutic agents are released. Indeed, achieving this objective would allow reaching higher local concentration near to the operative levels demonstrated in vitro, while reducing the overall administered dose (and consequently systemic toxicity associated). According to this objective, as a result of the research efforts developed in the last years, a diversity of internal and external factors can control the specific release of bioactive phytochemicals, according to a range of factors, including pH, the activity of local, temperature, ultrasound, magnetic field, and/or light incidence [184].

Moreover, recently, the nanoencapsulation of compounds identified as potential therapeutic agents has raised growing interest, due to the augment of the range of biomaterials with valuable applications in this field [163,185]. Thus, polymeric nanocarriers can increase the bioavailability, improve solubility, and prolong the shelf-life of potential compounds which, to date, have been difficult to deliver in a controlled way. For instance, oil-cored nanocapsules improve originally the administration of bioactive compounds, allowing targeted delivery and controlled, long-term, release that contribute to decrease the dosage and frequency of administration giving rise to an increased patient compliance [184]. Due to this, the layer-by-layer self-assembly of pH-sensitive building blocks has been explored as a promising approach to obtain biomaterials with customized properties [186] and, thus, with interesting applications as stimuli-responsive nanocarriers for controlled release of phytochemical compounds, which could also provide transport capacity through diverse biological barriers [187]. Moreover, the use of appropriate pH-dependent biocompatible polyelectrolyte for nanocapsule shell formation can provide the tools required to design non-toxic nanocarriers, with shell permeability [184].

Loading bioactive phytochemicals in nanoparticles turns bioactive compounds into more effective and contributes to achieve site-specific delivery. Once into the cells, some nanoparticles release the bioactive phytochemicals slowly, contributing to sustained therapeutic effects, which constitutes an additional advantage [188]. This capacity was demonstrated recently by using rhodamine-loaded polyethylene glycol-nanoparticles. Upon this characterization, after the application of rhodamine labeled nanoparticles to SH-SY5Y neuroblastoma cells, red fluorescence is visualized in the cytoplasm, suggesting that nanoparticles are internalized via endocytosis [189], saving the phospholipidic barrier that the cell membrane represent for hydrophilic compounds. An additional demonstration of these advantages was obtained on prostate cancer DU145 cells with similar positive results [190].

In addition, in relation with the administration of bioactive compounds, pursuing the development of activity at neural tissues and in the frame of deficits or overexpression of neurotrophins, responsible for neurodegenerative diseases and psychiatric disorders, to date, the research has demonstrated a delivery system competent to control of neurotrophin dosage in the brain. In fact, the major outcomes obtained in this issue have suggested that carrying bioactive compounds in nanoparticles might favor targeted delivery in specific brain areas, minimizing biodistribution to the systemic circulation and, consequently, toxic side-effects. Indeed, this approach provides valuable benefits concerning neuroregeneration [191].

In addition to oral administration, polymeric microspheres, scaffolds, and conduits have been used as sustained-release systems for neurotrophic proteins [192,193] that even provide enhanced neuroregeneeration, relatively to the implantation of polymeric scaffolds resorting to invasive surgery techniques into the central nervous system [191]. Indeed, this situation is exemplified by a slow release achieved of glial cell line-derived neurotrophic factor, by-using micro-reservoirs created by biodegradable poly-(lactic-*co*-glycolic acid) microspheres or by Poly(lactic-*co*-glycolic acid) microparticles [194,195].

Although the actual significance and advantages of the abrupt release of bioactive compounds, when using controlled delivery systems, has not been entirely ignored over the last years, to date, no plenary successful theories have explained completely the phenomenon. In addition, it has to be considered that the negative effects, associated to burst release, are pharmacologically dangerous and economically inefficient [196]. To overwhelm this inconvenience, new nanoformulations composed of thermo-sensitive gelling copolymer has been successfully formulated and characterized. The negligible amount of bioactive compounds released, when using these systems, prevent the toxicity enclosed to peaks and valleys of concentration in target tissues. Therefore, the delivery systems developed resorting to the application of this copolymer can minimize the side effects associated with frequent injections for the administration of therapeutics of interest, without reducing the efficiency of the bioactive compound, serving as a promising platform for avoiding pathophysiological complications [173]. However, the demonstration of such advantages is based on in vitro evidence, and remains to be further elucidated upon in vivo evaluations.

Studies on nanoencapsulation and controlled release from nanostructured carriers have demonstrated the safety of these systems and their contribution to achieve operative concentrations in target cells and tissues, when applying polymer- and lipid-based nanostructured systems [191]. Due to this and according to previous reports, on the capacity of such carriers to protect instable therapeutic proteins, e.g., from enzymatic degradation and other environmental stress factors [191], similar approaches could be applied to the administration of bioactive phytochemicals and take advantage of their biological potential, preventing their metabolization in vivo. In this frame, the research on the beneficial effects of anthocyanins loaded nanoparticles against neurotoxicity in vitro, has been focused on the evaluation of the cytotoxic profile of nanoparticles in the human neuroblastoma model (SH-SY5Y cell line). Upon this study, different concentrations of both free-anthocyanins and anthocyanins loaded in poly(lactide-*co*-clycolide) nanoparticles allowed discarding significant cytotoxic effects, while the joint application of anthocyanins loaded nanoparticles increased the viability of treated cells, by protecting them from neurotoxic events [168]. Thus, poly(lactide-*co*-clycolide) nanoparticles might constitute promising phytochemical carriers with negligible cytotoxicity events. Additionally, the administration of the mentioned nanoparticles provides the advantage of avoiding the need of removing an eventual implant placed as a local source of the bioactive compounds, and thus, no surgery would be required [197,198].

## 5. Toxicity Facts Associated to the Administration of Biodegradable Nanoparticles

In respect to the toxicity associated to the administration of biodegradable nanocarrier systems for bioactive compounds, as far as we are aware, there is no evidence on deleterious effects, although the risks to human health that could be associated to the long term use, remain under explored [148]. The mechanisms responsible for the deleterious effects of nanoparticles in the frame of complex biological systems are associated to increased productions of Reactive Oxygen Species (ROS) and free radicals, that could give rise to oxidative stress, inflammation, and consequently to several disabling pathophysiological situations, associated with solvent residues and polymers toxicity [199]. To shed some light on the actual toxicity of nanoparticles, it is required to evaluate the separate trials independently, paying attention to interactions between nanocarriers and biological systems. This way of processing the available information on the toxicity, associated to nanoparticles, is crucial to establish the reliability of the delivery systems. Actually, to translate successfully nanoformulations from the experimental level to their practical clinical application, it is essential to establish their safety profiles, including the evaluation of immunotoxicity. In this concern, special attention should be paid to the linkage between the physico-chemical and functional properties of nanoparticles (surface charge, size, modulate uptake and interactions with cells, control over surface modification and biodegradation of nanovectors), and the extent in which, these features, contribute to achieve the functional potential envisaged and to minimize potential health risks [200].

Hence, toxicological facts associated to nanocarrier systems involve several physiological, physicochemical and molecular considerations. However, despite the evident interest of understanding the toxicity of these delivery systems, to date, it has not been deeply explored, possibly because the industrial use of this technology is in its infancy. From the information available in the literature regarding this, it is noticed that toxicological facts associated to nanocarriers is closely linked to their size and shape, biomaterials, as well as to their capacity to cross biological barriers [201]. Regarding the molecular mechanisms, behind the toxicological effects, associated to treatments with bioactive compounds carried on biodegradable nanoparticles, it has been noticed the formation of pro-oxidants after their administration, disrupting the balance between their production and the detoxification capacity of cells. This fact also entails augmented inflammation reactions due to the level of redox-sensitive transcription factors [202,203].

In relation with the use of nanoparticles to carry bioactive compounds addressed to be ingested in foods, water or drug delivery devices, the interest of biodegradable nanoparticles and the constraints associated to their toxicity, following oral ingestion, has been associated to the occurrence of deleterious effects on liver, kidney and spleen. In addition, even if the mechanisms responsible for triggering the immune response induced by nanoparticles (especially concerning non-protein-based carriers) are not clear, there is a growing attention on possible allergic reaction [202]. To gain a further insight in this issue, further studies of the immunogenicity of nanocarriers are required to understand under which conditions they are identified by the immune system, deserving the generation of a specific immune responses [202]. Polyethylene glycol (PEG)-grafted liposome infusion has been described to trigger non-IgE-mediated signs of hypersensitivity [204]. On the other hand, advantages can be obtained from the immunogenic characteristics of nanoparticles, as these features suggest them as valuable adjuvants, for instance, for the development of vaccines.

## 6. Future Perspectives for Targeting and Controlled Delivery

In the coming years, the research trends in the application of edible nanoparticles to the administration of bioactive phytochemicals will be closely linked to new manufacturing strategies that combine multiple structural designs, more specific for the diverse types of bioactive compounds. Achieving this objective is foreseen to strengthen the property of nanoparticles and to combine the benefits identified on two or more types of biomaterials. This strategy would contribute to maximize the benefits of loading bioactive compounds on nanoparticles, in terms of effectivity, extended release, and in situ delivery control. Among the desired features, target delivery has not been completely developed yet, remaining an issue with future prospects for implementation. In this sense, materials and fabrication strategies would allow the creation of edible nanoparticles with improved properties is constantly ongoing. Once improved, the specificity and versatility of available nanoparticles, the administration of bioactive compounds orally, by using edible nanoparticles, will constitute a valuable approach that would allow to take advantage from the actual potential of phytochemicals to prevent and treat specific pathophysiological status, due to accurate tissue-targeted delivery.

One of the major constraints enclosed to the administration of bioactive compounds, even when using nanotechnology products, lies in the degradation of these compounds in the gastrointestinal lumen before being absorbed. Thus, although in vitro the works, available in the literature, reveal promising potential of the use of nanoparticles for the administration of phytochemicals loaded nanoparticles, nowadays it is required to complete the determination of the impact of gastrointestinal digestion, by informative in vitro simulation models, as cost-efficient tools for forecasting the oral bioavailability [167,205]. With this aim, in addition to implementing a model mimicking the salivary, gastric, and intestinal fluids concerning salt and enzyme concentrations, it is required to avoid an excessive simplification of the models, by including appropriate simulators of gastrointestinal dynamics, structure, and mechanical issues.

The consideration of gut metabolism, on the actual biological activity of compounds ingested by oral administration, requires a review of the current definition of “bioaccessibility” and “bioavailability”, emphasizing the value of the absorption in the upper gastrointestinal tract. Indeed, information on the features of this physiological process, according to the chemical properties of the bioactive compounds of interest, and the derivatives that can be formed during the gastrointestinal digestion, is crucial for the rational design of the specific nanoparticle system. With this objective, the selection of the type of nanoparticles, to improve the absorption at the gastric and small intestine (duodenum) level, may not serve as a gold standard anymore, because, for some phytochemicals, high bioefficacy is associated with the additional compounds synthesized, for instance in the large intestine, as a result of the metabolism of the local microbiota, turning into undesirable their absorption in the upper gastrointestinal.

Besides, when evaluating the effects of nanoparticles and/or nanovesicles on the bioefficacy of phytochemicals, special attention should be paid to eventual changes in the nanoparticle structure as a result of the digestive conditions. Indeed, this modification of the nanoparticles structure could entail changes in their functionality as carriers of bioactive nutrients and non-nutrients and, consequently, induce misunderstanding results. Thus, considering that, when mixed with digestion fluids, the dilution would have a crucial effect on the stability of micelles, liposomes, and nanoemulsions, is required. This deserves to be considered because the surfactant concentration should be maintained in a slim range. Moreover, pH and ions environments in the gastrointestinal fluids, as well as the enzymatic activity in these compartments could also compromise the stability of nanoparticles and the successful development of their role as carriers of bioactive compounds [122]. According to these constraints, it is necessary to evaluate the behavior of nanoparticles in complex matrices and biological systems that will provide actual information on their practical significance.

An additional situation, that needs to be addressed, is the extent in which nanoparticles modify the pharmacokinetics of bioactive phytochemicals, which would contribute to draw new applications, for instance as boosters of the internalization speed. Clarifying this situation is essential for the proper design of the sampling timing to determine pharmacokinetics bioavailability and bioactivity of bioactive compounds subject of study. Overall, encapsulated phytochemicals within appropriate nanoparticles enhance the bioavailability by protecting them from degradation during storage and gastrointestinal digestion, improving solubility in aqueous media, augmenting contact time with the intestinal wall, increasing of the mucus penetration and intestinal permeation, facilitating cellular uptake, prolonging residence time within the body circulation, controlling release rate and site, and altering, according to the envisaged results, the microbiota metabolism. In addition, it should be considered that loading bioactive compounds in nanoparticles, somehow, could be an interesting strategy to prevent their metabolic conversion, while to date, little is known about how the modification of cellular signaling routes differs between free and nanoparticles-linked phytochemicals. In this regard, seems evident that the interaction between phytochemicals and matrix material, or molecules in complex biological systems (for instance in mammals), deserves to be further explored concerning metabolism and bioefficacy [122]. Hence, the information retrieved from studies, focused on the correlation between the application of bioactive compounds carried by nanoparticles with the metabolic profile, biodistribution and bioactivity, will be very useful as a guide for future evaluation of the bioefficacy and predictable in vivo properties.

Concerning the final intestinal stage, the interactions between phytochemicals and drugs with gut microbiota, as well as the consequences of these interactions on bioefficacy, remain underexplored, even if it seems evident that they constitute additional challenges. Indeed, the application of the gathered knowledge on the use of nanoparticles, as carriers of bioactive phytochemicals, would provide the opportunity to decode and take advantage of the reciprocal interactions between phytochemicals and gut microbiota.

Despite the rational utility of the nanotechnology to enhance the bioavailability and bioactivity of bioactive compounds, nanoparticles are synthesized by physical and chemical methods, by using expensive and hazardous chemicals, especially concerning metallic biomaterials, which could limit their actual application in vivo. These constraints are also enclosed to the environmental impact derived of the residues, generated from the nanoparticles synthesis, being required green technologies using no toxic reagents to prepare metal nanoparticles. In this regard, the synthesis of nanoparticles using eco-friendly and biocompatible reagents could contribute to minimize the side effect of these processes.

## 7. Conclusions

Nutraceuticals’ potential benefits have been demonstrated, but increasing applications in the near future, for the prevention of diseases onset and severity, are highly demanded. To take maximum advantages of bioactive compounds (nutrients and non-nutrients), responsible for the functionality of nutraceuticals, promising applications have been suggested based on combinations of individual, well characterized, bioactive phytochemicals. This approach would allow synergic situations that would help to reduce dosages and, thus, eventual toxic side effects, while becoming instrumental in the prevention of an array of pathophysiological situations.

To improve the bioavailability of phytochemicals identified as valuable bioactive compounds, in the frame of a specific clinical entity of health disorders, nanoparticles have been investigated as promising delivery systems that could contribute to control release and to fine-tune pharmacokinetics, bioavailability, and bioefficacy. Indeed, nanotechnology would contribute to improve stability of encapsulated bioactive nutrients and non-nutrients against environmental changes (in the diverse in vivo environments) and to release control. Therefore, it should be assumed that nanoparticles have great potential as phytochemical carriers, as, at the dosages monitored, they are not cytotoxic. However, critical aspects and potential for application of nanotechnology in Food Science and Technology field, should be noticed, especially regarding the implementation of green technology for their development and their use in the studies of additional issues, such as the crucial role of the intestinal microbiota for obtaining additional bioactive metabolites, contributing to the health benefits attributed to plant foods and nutraceuticals.

## Abbreviation

EDC	1-(3-(dimethylamino) propyl) 3-ethylcarbodiimidehydrochloride
